# Uncertainty-Aware Zero-Day Botnet Detection for IoT Networks with Real-World Edge Deployment on Resource-Constrained Hardware

**DOI:** 10.3390/s26185863

**Published:** 2026-09-16

**Authors:** Cinmoy Purkaystha, Yesha Nilesh Gandhi, Prashant Kumar

**Affiliations:** Department of AI and Big Data, Woosong University, Daejeon 34606, Republic of Korea; purkaysthac@gmail.com (C.P.); yeshagandhi2525@gmail.com (Y.N.G.)

**Keywords:** IoT sensor networks, botnet detection, zero-day attack detection, Monte Carlo dropout, explainable AI, XGBoost

## Abstract

Closed-set evaluation is the most common method in Internet of Things (IoT) sensor network botnet attack detection research. In this setting, classifiers are tested only on attack families encountered during training. As a result, they often fail to detect genuinely new (zero-day) malware in real-world environments. This study evaluates a calibrated two-tier detection pipeline combining an XGBoost gradient-boosted tree classifier for known-class prediction with a Monte Carlo (MC) dropout multilayer perceptron (MLP) as an independent uncertainty estimator, under conditions closer to real deployment than most prior evaluations. The uncertainty model is used to flag unfamiliar traffic that may represent zero-day traffic. The classifier is trained and calibrated on the Bot-IoT dataset and evaluated for zero-day generalization on N-BaIoT, which contains the previously unseen Mirai and BASHLITE malware families. During data preparation, a severe train/test duplication problem was identified. Around 97% of a naive split was found to be duplicates and was removed before splitting. The base classifier achieved 99.4% accuracy in known-class detection. However, the confidence scores were negatively correlated with zero-day traffic, producing an area under the receiver operating characteristic curve (AUROC) of 0.32. This indicates the overconfident misclassification of unseen attacks. The proposed uncertainty gate improved zero-day discrimination to an AUROC of 0.66, with catch rates reaching 13.75% for Mirai. An explainable AI analysis using Shapley Additive Explanations (SHAP) linked this gap to packet size statistics. To evaluate practical feasibility, the complete framework was deployed on an embedded Raspberry Pi Compute Module 5 (CM5) edge device. The full uncertainty-gated pipeline achieved mean inference latency of 15.03 ms per sample. Overall, these findings demonstrate that uncertainty-aware gating can improve zero-day robustness over conventional closed-set classification in resource-constrained IoT sensing environments.

## 1. Introduction

The rapid expansion of the Internet of Things (IoT) has introduced an increasingly large attack surface for network security. IoT devices are widely used as consumer and industrial IoT devices. Many of them function as the sensing endpoints of these networks and are often deployed with weak default credentials, outdated firmware, and limited built-in security controls. This makes them easy targets for attacks, which can compromise a large number of devices [1] and degrade their integrity. A compromised device becomes a platform from which an attacker can act against the wider sensor network and the systems that depend on it. The infected devices are commonly recruited into botnets, which coordinate distributed denial of service (DDoS) attacks, reconnaissance, and data exfiltration campaigns at scale. To address this emerging threat, machine learning (ML)-based intrusion detection systems (IDSs) have become widely popular for identifying botnet traffic in IoT networks because of their ability to learn complex traffic patterns without manually specified signatures.

However, most of the published IoT botnet detection studies have been conducted under closed-set conditions. In this setup, a classifier is trained and tested only on attack families that already exist in the training data. Recent work illustrates this pattern clearly. For example, an ensemble framework from 2025 that combined convolutional and recurrent architectures with classical classifiers reported 100% accuracy on the Bot-IoT dataset under this closed-set protocol [2]. These results confirm that known attack families can be separated well when sufficient features are available. Nevertheless, such studies do not determine the behavior of a deployed system when it encounters a completely new botnet family—a situation known as a zero-day attack.

A key limitation of standard softmax-based classifiers is that they cannot distinguish between known and unknown inputs. Softmax output probabilities always sum to one across the known classes. Thus, even when an input does not resemble any known class, the model produces a confident prediction for the closest matching class. This overconfidence problem is well documented in the machine learning literature and motivates the use of explicit uncertainty quantification. Monte Carlo (MC) dropout is one such method, which approximates Bayesian model uncertainty by keeping dropout active at inference time and aggregating the variance across repeated stochastic forward passes [3].

Another important challenge is cross-dataset generalization, which is rarely evaluated rigorously. A recent heterogeneous benchmark proposed a shared feature vocabulary spanning multiple IoT and general network intrusion datasets. It found that models trained on one dataset and evaluated on another often exhibit a significant drop in performance, with F1 scores falling to 0.40–0.56 [4]. Their own vocabulary reached only about 15% feature coverage when applied to Kitsune-style statistical datasets such as N-BaIoT. This occurs because it assumes forward and backward directional flow information, which these extractors do not provide. Thus far, cross-dataset zero-day evaluation, uncertainty-based abstention, and real embedded-hardware validation have each been studied separately but not in combination.

This work addresses this gap with a two-tier detection framework. A gradient-boosted tree classifier performs known-class prediction. On the other hand, a separate MC dropout multilayer perceptron (MLP) estimates predictive uncertainty and flags traffic outside the training distribution as a possible zero-day attack. The framework is trained on the Bot-IoT dataset and evaluated for zero-day generalization on N-BaIoT, which is built with a different extraction methodology. N-BaIoT contains malware families (Mirai and BASHLITE) that are structurally distinct from those in Bot-IoT. During this work, we identified a severe train-and-test duplication artifact affecting 97% of a naively constructed split. This type of leakage can inflate the reported performance considerably and could affect other studies that use similarly structured IoT flow datasets. Therefore, we corrected it by deduplicating the data before splitting. We further deployed the complete two-tier pipeline on a Raspberry Pi Compute Module 5 (CM5) (Raspberry Pi Ltd., Cambridge, UK), a resource-constrained embedded platform representative of real IoT edge gateways, and report the measured inference latency and memory footprint rather than simulated feasibility. The central contribution of this work lies in how this pipeline is calibrated and evaluated under conditions that most prior studies in this area do not test, rather than in the individual components themselves.

The contributions of this work are as follows:

1. We identify and correct a severe train/test duplication artifact in a widely used IoT botnet dataset split and show that it inflates the reported accuracy from a realistic level to near 100%.
2. We combine a gradient-boosted tree classifier with an independently trained MC dropout uncertainty gate and calibrate the gate against a target false positive rate on known traffic, with an emphasis on the calibration and evaluation methodology.
3. We evaluate zero-day generalization across two IoT botnet datasets built with different extraction methodologies, rather than through a simulated held-out class from a single dataset.
4. We deploy the complete pipeline on a Raspberry Pi CM5 and report the measured latency and memory rather than claimed feasibility.

The remainder of this paper is organized as follows. Section 2 reviews related work on zero-day and cross-dataset IoT botnet detection. Section 3 describes the datasets, feature alignment methodology, classifier architecture, and calibration steps. Section 4 presents the classifier comparison, zero-day detection results, feature attribution analysis, and edge deployment measurements. Section 5 discusses the implications and limitations of these findings, and Section 6 concludes the paper.

## 2. Related Work

Prior work on IoT botnet detection can be divided into three themes: closed-set classification, zero-day and cross-device generalization, and heterogeneous cross-dataset benchmarking.

Closed-set classification remains the dominant evaluation protocol. In addition to the ensemble framework discussed in Section 1 [2], according to recent studies, most machine learning-based IDS studies report accuracies above 99% when tested on attack families already present during training [1]. These results are valid, but they do not reflect how a classifier behaves against traffic that it has never seen before. This is the central question for practical deployment.

A related line of work has examined open-set recognition for industrial network intrusion detection more broadly. Yu et al. combined gated recurrent units with prototype learning to separate known and unknown attack classes, reporting strong known-class accuracy alongside the effective detection of unknown intrusions [5], although their method was evaluated on industrial control traffic rather than IoT sensor network botnet traffic and the study does not address cross-dataset generalization or embedded hardware deployment. Few works have attempted zero-day generalization directly. Popoola et al. [6] proposed a federated deep learning framework evaluated on the Bot-IoT and N-BaIoT datasets—the same two datasets used in the present study. Their study reported that federated aggregation across simulated edge devices improved the detection of previously unseen attack patterns compared to centralized training. However, they do not report a calibrated false positive rate on known traffic, and their architecture was not deployed or measured on physical embedded hardware. In a related automotive IoT setting, Amara Korba et al. [7] proposed a meta-ensemble of Isolation Forest models combined through particle swarm optimization and reported a zero-day detection rate of 77.32%, against 92.80% for known attack families. This gives a useful reference point for the scale of zero-day performance that unsupervised anomaly detection can achieve. However, their system was evaluated on a single automotive botnet dataset instead of a cross-dataset setting consisting of different collection methodologies. Moreover, they do not report deployment on resource-constrained hardware.

A recent online learning approach evaluated an adaptive k-nearest neighbor method with self-adjusting memory for zero-day attack detection across IoT and industrial IoT settings, reporting a detection rate of 1.00 on simulated zero-day attacks while maintaining a very small memory footprint [8]. This demonstrates the feasibility of lightweight, deployment-aware zero-day detection, although the evaluation relied on simulated rather than genuinely cross-dataset zero-day traffic and did not include uncertainty quantification. Separately, a heterogeneous deep stacked ensemble combining GRU, LSTM, DNN, and MLP base models was proposed to improve cross-domain generalization across four NetFlow-based intrusion detection datasets [9], reporting substantial F1-score improvements over prior cross-domain methods. This work shares our study’s interest in ensemble-based robustness across heterogeneous datasets, but it targets general network intrusion detection rather than IoT botnet zero-day detection specifically, and it does not report embedded hardware deployment.

Heterogeneous cross-dataset benchmarking has recently been proposed as a more direct way to address generalization. Sarhan et al. [10] evaluated NetFlow and CICFlowMeter feature sets across three network intrusion datasets, including Bot-IoT, and used SHAP to explain which features are common across environments. Their analysis remains within closed-set classification and does not consider zero-day detection or uncertainty-based abstention. As discussed in Section 1, a more recent canonical feature vocabulary present in multiple IoT and general network intrusion datasets showed a substantial cross-dataset performance loss, with F1 scores as low as 0.40–0.56 [4]. This study evaluated generalization accuracy directly rather than through an uncertainty-based abstention mechanism, and its hardware measurements were obtained on a server-class graphics processing unit (GPU) instead of a resource-constrained embedded device.

Overall, existing works have explored zero-day generalization, uncertainty-based abstention, and embedded hardware deployment largely in isolation. This study brings all three together, with a calibrated uncertainty gate evaluated across datasets built with different extraction methodologies, deployed and measured on physical embedded hardware representative of real IoT edge gateways.

## 3. Materials and Methods

The overall research design follows three deliberate choices, each addressing a specific gap identified in Section 2. First, known-class classification and zero-day detection are handled by two independently trained models rather than a single combined model, since a classifier trained only to classify known traffic has no mechanism to express when an input does not belong to any known class at all (detailed in Section 3). Second, zero-day generalization is evaluated across two datasets built with genuinely different collection methodologies, rather than through a simulated held-out class within a single dataset, so that the evaluation reflects a real distributional shift rather than an artificial one. Third, all performance and latency claims are founded directly on physical embedded hardware, rather than being estimated or simulated, so that the reported feasibility reflects actual deployment conditions.

### 3.1. Datasets

Two publicly available IoT botnet datasets were used in this study. They were selected specifically because they were collected using different methodologies and contain malware families that are structurally distinct from one another. This allows us to run a genuine zero-day evaluation with unknown attack families. This is different from using a simulated held-out class taken from a single dataset that shares the same underlying collection process as the training data.

Bot-IoT [11] was collected on a synthetic testbed at UNSW Canberra. Both normal and botnet traffic was generated in a controlled network environment using a custom in-house attack toolkit, and flow-level features were extracted using the Argus network auditing tool. The dataset includes raw flow information such as the source and destination IP addresses, source and destination ports, and transport protocol. It also contains derived statistics like the packet count, byte count, and inter-arrival rate. The full dataset is larger than 72 GB when raw packet captures are included. For this reason, we used the 5% extracted subset recommended by the authors for computational tractability. This subset retains the full label structure and comprises 3,668,522 rows across five categories: distributed denial of service (DDoS), denial of service (DoS), reconnaissance, theft, and normal.

N-BaIoT [12] was constructed by infecting nine commercial IoT devices with real Mirai and BASHLITE malware (labeled gafgyt in the dataset’s own files; we use BASHLITE throughout the text and retain the native label in figures generated directly from the data), covering doorbells, thermostats, security cameras, and a baby monitor. Unlike Bot-IoT, N-BaIoT extracts 115 statistical features per traffic window using a Kitsune-style stream feature extractor. These features summarize packet size and timing statistics at five aggregation scopes, ranging from individual source–MAC–IP pairs up to aggregated host pair channels, each computed across five exponentially decayed time windows. This means that N-BaIoT contains no IP addresses, ports, or protocol fields but merely derived statistical summaries. We used the full dataset, which totals 7,062,606 rows across benign traffic and ten Mirai and BASHLITE attack subtypes.

The methodological difference between these two datasets is what enables the zero-day evaluation in this study. Bot-IoT’s attack traffic was generated with a synthetic, in-house botnet toolkit built specifically for the UNSW testbed, whereas N-BaIoT’s attack traffic was produced by real, publicly circulated Mirai and BASHLITE malware infecting commercial hardware. These are not variants or subcategories of one another. Training on Bot-IoT and evaluating on N-BaIoT requires the model to generalize to malware behavior that it has never seen, which more closely reflects the conditions faced by a deployed zero-day detection system in a real IoT environment.

### 3.2. Feature Alignment

A significant practical problem when combining these two datasets is that they share no overlapping raw column names. This is not merely a labeling inconsistency. It arises from their fundamentally different feature extraction approaches. Bot-IoT represents each network flow individually with raw identifiers and per-flow statistics. On the other hand, N-BaIoT represents traffic as decayed statistical summaries over time windows with no notion of an individual flow or identifier. Thus, any attempt to combine these datasets for a single model requires a shared feature space to be built from scratch, rather than relying on any existing column overlap.

Bhilwarawala et al. proposed a cross-dataset alignment methodology for heterogeneous IoT and network intrusion datasets [4]. The feature extraction method of our study was motivated by this technique. The methodology rests on three principles. First, a feature is outlined between datasets only when a genuine physical equivalence can be justified, not by inventing a proxy feature simply to fill a slot in a fixed-size vector. Second, when no genuine match exists for a given dataset, the feature is explicitly zero-filled for that dataset instead of being approximated with a loosely related one. Third, the resulting feature coverage for each dataset is fully disclosed, rather than being presented as if every dataset contributed complete information.

We did not directly use the 46-feature standard vocabulary proposed in the benchmark. Their vocabulary is based on forward and backward directional flow features generated by tools such as CICFlowMeter. These tools are known to assume a bidirectional flow model with clearly distinguishable source and destination sides. In contrast, N-BaIoT uses a Kitsune-style extractor that has no such directional concept. The benchmark evaluation confirms this mismatch, reporting only approximately 15% feature coverage for N-BaIoT under the proposed vocabulary. Bhilwarawala et al. [4] acknowledge that this limitation led them to place less emphasis on N-BaIoT in later stages of their pipeline.

Instead, we built our vocabulary around a property that both datasets genuinely share, namely multi-window traffic statistics for each source entity. Bot-IoT already contains window-based aggregates for each source IP and protocol, including the total bytes, total packets, and connection counts. N-BaIoT provides similar information through its host-level and source-level decayed statistical features at multiple time constants. Focusing on this shared structure, rather than around either dataset’s native schema, allowed us to construct a common nine-feature representation. This prevented equivalences that do not exist. The resulting vocabulary, along with the corresponding source column or derived feature used for each dataset, is presented in Table 1.

Two features required estimation because a direct equivalence was missing. First, pkt_size_var_fast in Bot-IoT is approximated using the source and destination byte counts. Bot-IoT does not expose a true per-flow packet size variance. Second, pkt_size_var_slow is set to zero for Bot-IoT, since the dataset contains no comparable windowed packet size variance at the per-source IP level. Both limitations are acknowledged explicitly here to maintain transparency, following the disclosure practice recommended by Bhilwarawala et al. [4].

### 3.3. Data Deduplication and Splitting

An initial stratified split of Bot-IoT revealed a serious duplication problem. In total, 97.02% of the test samples were exact duplicates of feature vectors already present in the training set. The duplication was specifically concentrated in the flood-style DDoS (98.65%) and DoS (97.88%) categories. Only 8.81% of the training set found consisted of unique feature vectors. This caused the preliminary classifier accuracy to reach almost 100%, reflecting the memorization of repeated flow records instead of genuine generalization. This observation is consistent with the closed-set accuracy levels reported in previous Bot-IoT studies [2]. It also suggests that undetected duplication may affect other studies using similarly structured IoT flow datasets.

We corrected this issue by deduplicating samples before performing any train/test splitting. The combination of the nine standard features and the category label for both datasets was used in the deduplication process. This reduced Bot-IoT’s row count from 3,668,522 to 255,104 unique rows. The theft category contained only 79 rows afterwards. For the multi-class evaluation, these samples were merged with the reconnaissance category. Since both categories represent low-volume, non-flooding attack types that are distinct from the high-volume DDoS and DoS categories, this grouping did not change the underlying binary attack labels used in the detection framework.

**Figure 1:**
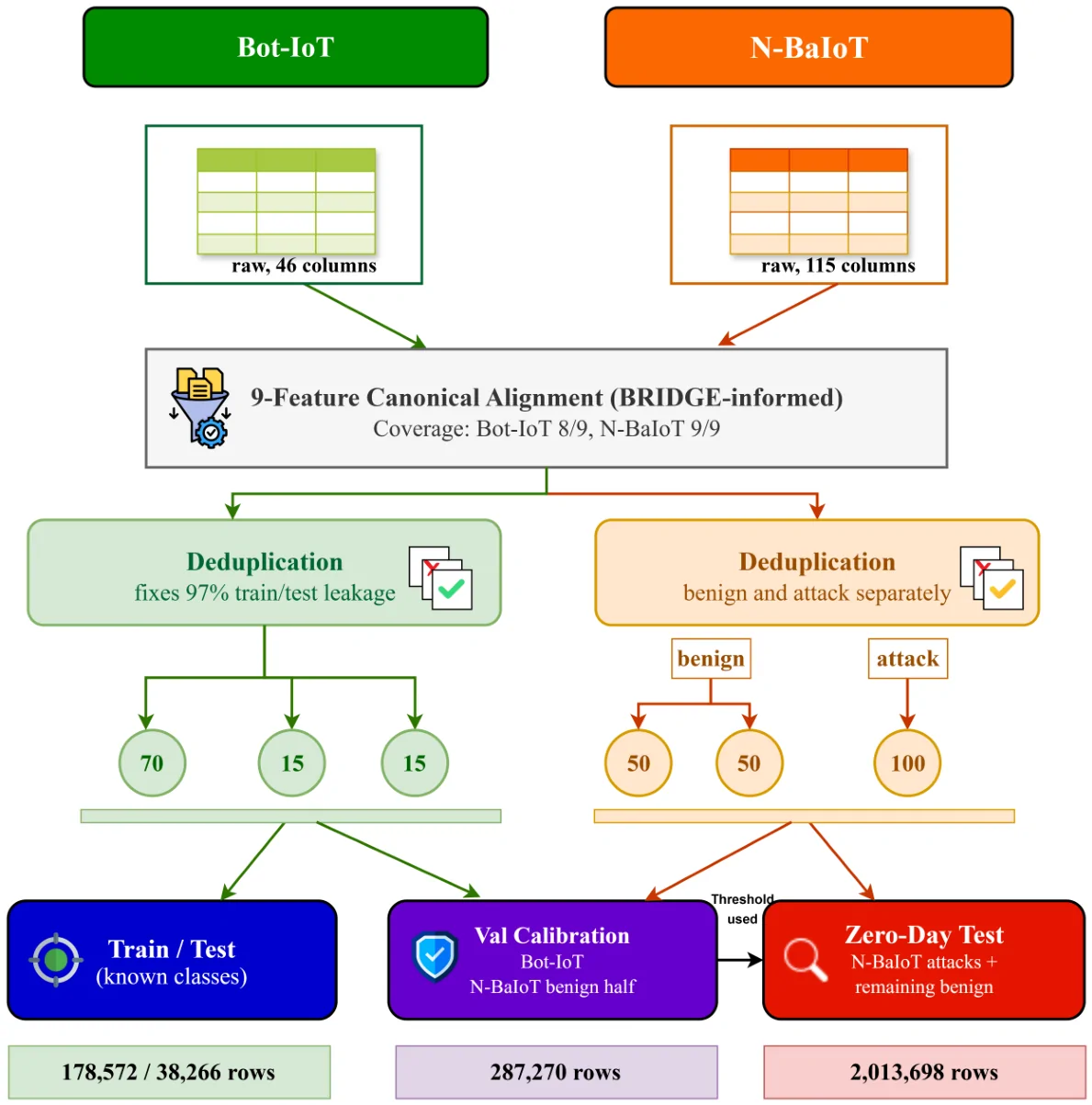
Data pipeline.

The deduplicated Bot-IoT data were divided into 70% training, 15% calibration–validation, and 15% test sets. The split was stratified using the grouped category labels to preserve the class distribution across the three partitions. N-BaIoT’s benign traffic, after deduplication, was split 50/50. One half was added to the calibration–validation set to improve false positive rate estimation on benign traffic. This was necessary since Bot-IoT’s normal category contained only 443 rows before deduplication, being too small to calibrate a false positive threshold reliably on itself. The other half remained in the zero-day test set. All N-BaIoT attack rows (Mirai and BASHLITE, deduplicated) remained entirely unseen during training and calibration; only benign traffic was used to supplement the calibration, so the zero-day evaluation was not exposed to any unseen attack samples. The final dataset splits are summarized in Table 2. Figure 1 illustrates the full pipeline described above, including the two raw datasets, canonical feature alignment, deduplication, and the final split into training, calibration, and zero-day test sets.

### 3.4. Two-Tier Detection Architecture

The primary challenge that this study addresses is that a standard classifier cannot distinguish between a confident correct prediction and a confident incorrect one. This is because, in a softmax classifier, the output probabilities are normalized across the known classes. Thus, a network flow that does not resemble any of them still receives a full probability distribution over the classes that the model does know. Moreover, the model has no mechanism to indicate that none of them may belong to an unknown class. A single classifier alone therefore cannot serve as a zero-day detector, regardless of how accurate it is on known classes. The proposed framework addresses this by separating the two tasks entirely. One model forms the prediction, and a second, independent model decides whether this prediction should be trusted.

**Figure 2:**
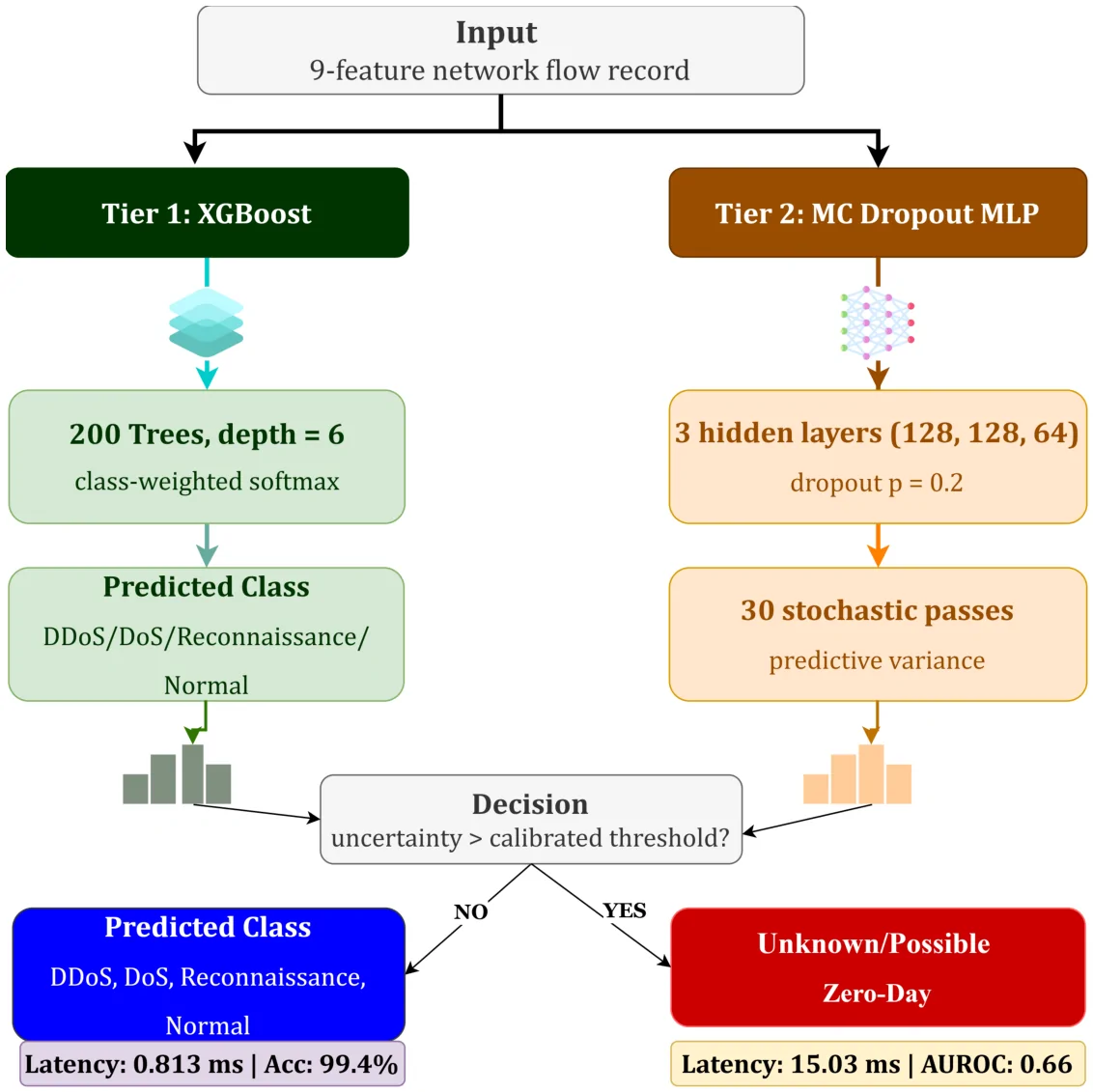
Two-tier detection architecture.

The framework consists of two independently trained models operating on the same nine-dimensional canonical feature vector, illustrated in Figure 2. Both models receive the same input at inference time, and their outputs are combined only at the final decision step.

Tier 1 performs known-class prediction using a gradient-boosted tree classifier, XGBoost [13]. The model was trained with 200 trees, a maximum depth of six, and a class-weighted loss function to reduce the effects of the remaining class imbalance among known categories. In order to justify the selection of XGBoost by direct comparison, five additional classifiers were trained under identical conditions on the same 178,572-row training set. These were LightGBM, random forest, a single unensembled decision tree, logistic regression, and Naive Bayes. The results are reported in Section 4. Support vector machines and k-nearest neighbors were excluded as they both scale poorly beyond approximately $10^{5}$ rows and would require subsampling, which would have rendered the comparison unfair with regard to the other five classifiers.

**Figure 3:**
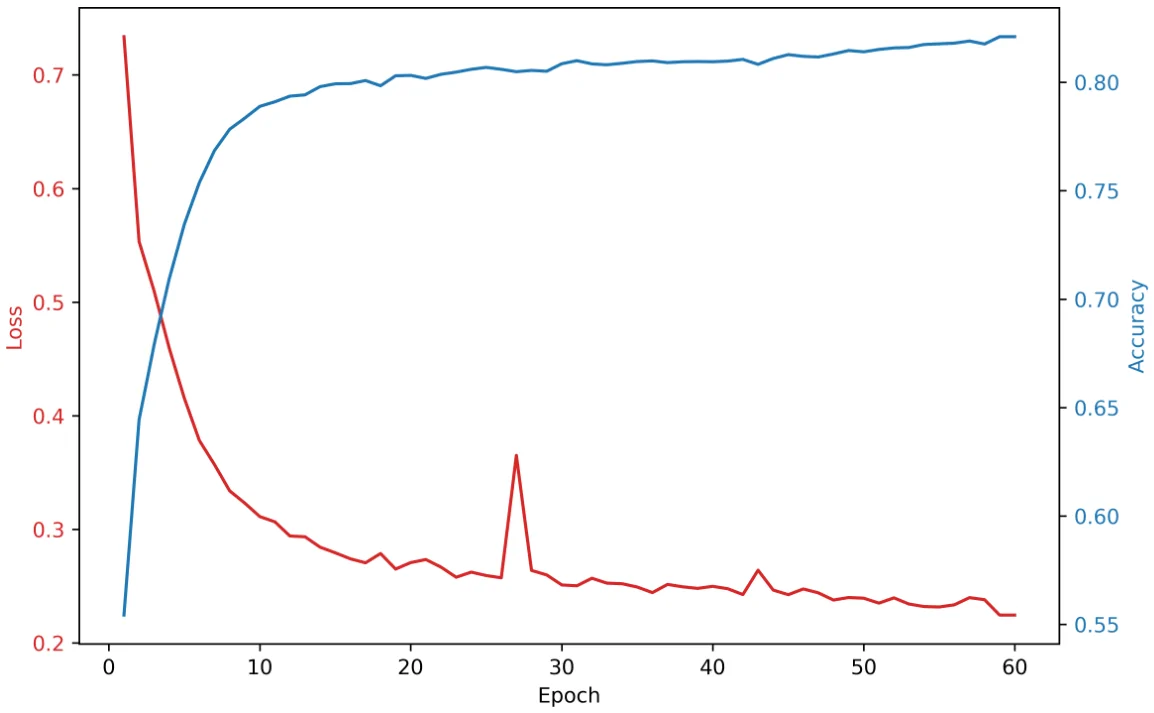
MC dropout MLP training curves.

Tier 2 estimates the predictive uncertainty using an MC dropout multilayer perceptron [3]. The network contained three hidden layers of 128, 128, and 64 units and a dropout rate of 0.2 and was trained for 60 epochs with the class-weighted cross-entropy loss and the Adam optimizer. Unlike standard neural network inference, MC dropout remains active and performs 30 independent stochastic forward passes for each input during inference. When an input resembles the training distribution, the outputs are barely effected by the random dropout masks and therefore remain highly consistent. In contrast, when an input is far from anything that the network has seen previously, the predictions become inconsistent and vary more across the forward passes. This occurs because the network has no stable learned response for this region. The variances in the predicted class probability estimates are then averaged across the four output classes. This variance is the uncertainty score used for the zero-day decision, not on the class predicted by Tier 2 itself. Figure 3 shows the training loss and accuracy of the MC dropout MLP over the 60 training epochs, confirming the stable convergence.

The two tiers are trained entirely independently on the same data, with both models remaining unaware of the other’s parameters, predictions, or representations. Tier 1 is optimized purely for known-class classification and Tier 2 purely for a well-behaved uncertainty signal. Training a single model to perform both tasks at once would risk degrading the performance of each task, which is why we chose to separate them.

### 3.5. Threshold Calibration

An uncertainty score alone is insufficient to constitute a decision. A threshold is required to convert a continuous score into a binary outcome and determine whether to trust the classifier’s prediction or flag the input as a possible zero-day attack. The threshold determines the trade-off between false positives and zero-day detection sensitivity. A lower threshold catches more unseen attacks but also flags more legitimate traffic as suspicious. A higher threshold reduces false alarms but may miss more zero-day attacks. Because real zero-day attacks are not available during deployment preparation, the threshold must be determined using only known traffic.

The threshold was calibrated using the calibration–validation set described in Table 2. This contains exclusively known traffic, including Bot-IoT’s known attack categories and its normal class, as well as the held-out half of the benign traffic of N-BaIoT. The additional N-BaIoT benign samples were added because Bot-IoT’s own normal category was judged to be too small on its own, at only 443 rows before deduplication. This number is not sufficient to support a statistically reliable false positive rate estimate. We computed the MC dropout uncertainty score for every sample in the calibration–validation set; we then set the threshold to the value corresponding to the 95th percentile of this score distribution. With this choice, exactly 5% of the known traffic falls above the threshold and would be incorrectly flagged as unknown. We consider this an acceptable operating point balancing detection sensitivity against the false alarm rate. This calibration step was performed once using only validation data, and the resulting threshold was then applied unchanged to the zero-day test set. The threshold was never adjusted using the zero-day test results, which would have invalidated the evaluation.

### 3.6. Comparison Methods

Two additional methods were evaluated alongside the MC dropout gate. Each method was designed to test a different possible source of zero-day detection signals.

The first was Isolation Forest [14], which was trained in a fully unsupervised manner on known-class training data only, without any class labels during training. Isolation Forest detects anomalies differently from MC dropout. It isolates outliers based on how quickly a data point can be separated from the rest of the data by random recursive partitioning, rather than through the variance of a supervised classifier’s stochastic predictions. It allows the zero-day detection results to be compared against a method that requires no calibrated classifier but only the raw feature distribution of known traffic. In order to ensure that the anomaly scores were directly comparable to the MC dropout gate, they were calibrated against the same 5% target false positive rate on the same calibration–validation set.

The second was a combined-signal variant. It was constructed to test whether Tier 1’s own prediction confidence, defined as one minus the maximum softmax probability across the known classes, provides useful information for zero-day detection beyond Tier 2’s produced uncertainty score. Z-score normalization was used to combine the two signals, with each using statistics computed from the calibration–validation set. The normalized values were then added together to produce a single combined score. This score was calibrated using the same percentile procedure described in Section 3.5. This variant was used as an ablation strategy. If Tier 1’s own confidence provides reliable information about novel traffic, combining it with Tier 2’s uncertainty should improve zero-day detection. However, if Tier 1 is overconfident and misleading on zero-day traffic, which is the main concern in this study, combining the two signals should degrade detection rather than improve it.

### 3.7. Feature Attribution Analysis

A calibrated uncertainty score explains how confident the gate is but does not explain why. To understand which of the nine canonical features actually drove the gate’s decisions, we computed Shapley Additive Explanations (SHAP) [15] for the Tier 1 classifier. We used the TreeExplainer implementation, which computes exact Shapley values for tree ensembles without the sampling approximation commonly used for non-tree models.

Instead of reporting only a global feature importance ranking, we compared feature attributions for two specific groups of samples: known-class traffic that the gate correctly accepts with low uncertainty and zero-day traffic that the gate correctly flags as unknown. This comparison highlights the features whose contributions change specifically when the model encounters previously unseen traffic, rather than which features matter for classification in general. Both known-class and zero-day samples were drawn from held-out test data not used in model training. Moreover, each group was limited to 5000 samples to keep the computational burden manageable, because the cost of exact SHAP analysis scales with both the sample count and tree depth.

### 3.8. Edge Deployment

A model that performs well in evaluation is not necessarily deployable. In order to examine practical feasibility, the complete two-tier pipeline was deployed on a Raspberry Pi Compute Module 5 (CM5), an ARM Cortex-A76 processor representative of real IoT edge gateways.

Both models were deployed using their native training frameworks, namely XGBoost for Tier 1 and PyTorch for Tier 2. We did not convert them to a common intermediate representation such as ONNX. This choice was deliberate rather than a matter of convenience because, in standard ONNX inferences, dropout layers are converted into a deterministic identity operation during inference. Because ONNX is designed for repeatable inference and not stochastic sampling of the same input, exporting the MC dropout MLP through this path would silently disable the production of the uncertainty estimations, without raising any error. As a result, every one of the 30 stochastic passes would return an identical result. Deploying both models through their native frameworks avoids this failure mode entirely, but at the cost of a larger runtime footprint on the embedded device than a quantized ONNX model would require.

We measured the per-sample inference latency under two conditions: Tier 1 only and the complete two-tier pipeline with all 30 MC dropout passes included. Both were measured using wall-clock timing over the same 2000 samples from the zero-day test set and transferred to the device. This ensured that the difference between the two measurements reflected specifically the additional computational cost of uncertainty estimation, not differences in input data. Peak memory usage was measured as the resident set size (RSS) reported by the operating system during the full pipeline run.

### 3.9. Evaluation Metrics

Different aspects of the evaluation require different metrics, since known-class classification and zero-day detection are fundamentally different tasks with different failure modes.

As reported in Section 4, the performance of the known-class classifiers was evaluated using the accuracy and macro-averaged F1 score, and they were compared directly against each other. The accuracy alone is misleading because Bot-IoT still contains class imbalance for known categories after deduplication. A classifier could achieve high accuracy by performing well only on the majority DDoS and DoS categories while failing on reconnaissance or normal traffic. The macro-averaged F1 score weights each class equally regardless of its size, so it more directly reflects whether a classifier has learned all known categories or only the most frequent ones.

Four complementary metrics were used together to evaluate the zero-day detection performance without any misleading results. The zero-day catch rate reflects the proportion of true attacks that are correctly flagged. However, this can be artificially increased by flagging everything as unknown, so it is always paired with the false positive rate on held-out benign traffic. Together, these describe the practical trade-off that a deployed system faces. We also report the AUROC and area under the precision–recall curve (AUPRC). These metrics were computed using the binary attack-or-benign label and the continuous uncertainty score instead of the thresholded decision. The AUROC summarizes the performance across all possible thresholds, not just the calibrated one. On the other hand, the AUPRC is reported alongside it because the zero-day test set contains more attack traffic than benign samples, which can cause the AUROC to appear more optimistic than the true precision-based performance.

All models were implemented in Python (version 3.11) using scikit-learn (version 1.5.0), XGBoost (version 2.0.3), LightGBM (version 4.3.0), and PyTorch (version 2.3.1). Complete implementation details and configuration files are available in the code repository referenced in the Data Availability Statement.

## 4. Results

This section presents the known-class classification performance, the zero-day detection results, a comparison with alternative detection methods, the feature attribution analysis, and the edge deployment performance on the CM5.

**Figure 4:**
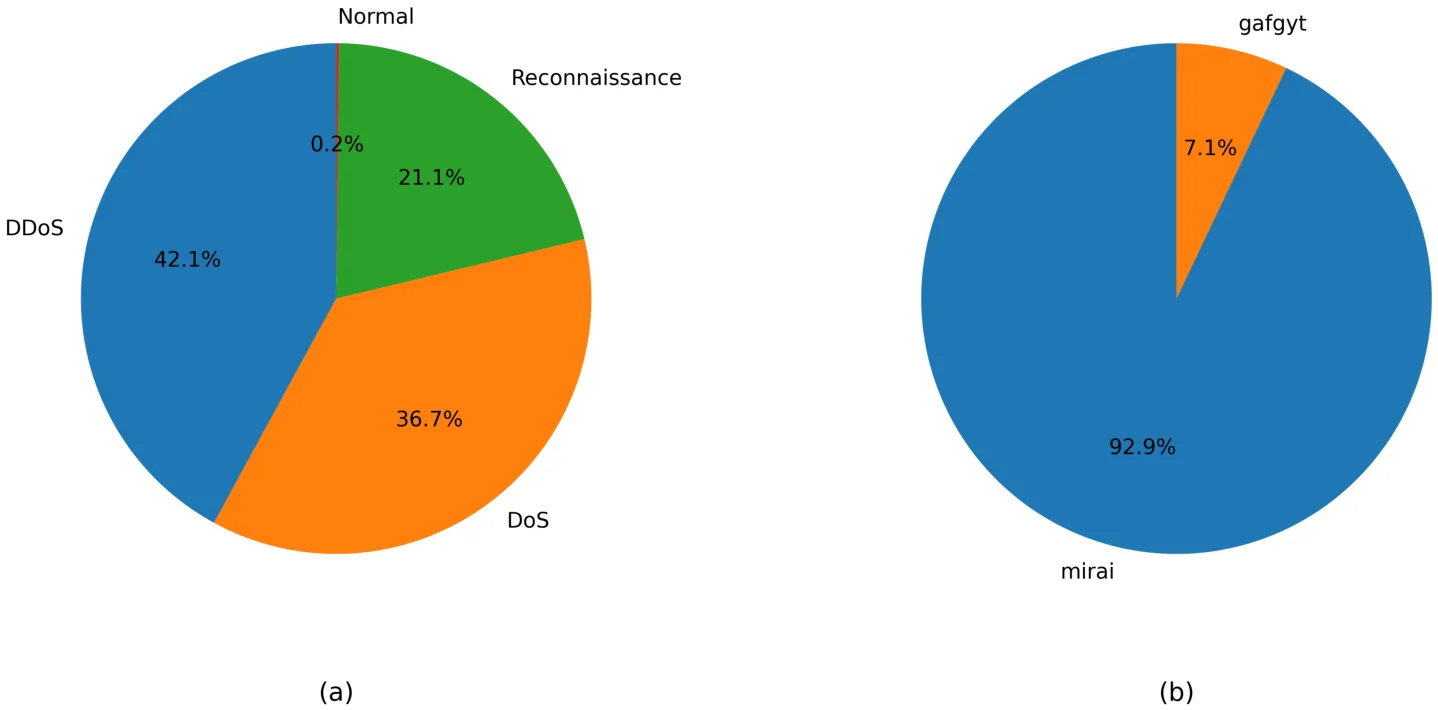
Class distributions after deduplication. (**a**) Bot-IoT known-class training data. (**b**) N-BaIoT zero-day attack families.

Figure 4 shows the class distributions of the two datasets after deduplication. After deduplication, the Bot-IoT training data remain dominated by flood-style attacks. DDoS and DoS together account for 78.8% of samples, while reconnaissance accounts for 21.1% and normal traffic for only 0.2%. This imbalance remains even after deduplication because it reflects the actual distribution of the traffic in the dataset, not an artifact of duplicate rows. This is the reason that class weighting was chosen over oversampling during training, as described in Section 3. N-BaIoT’s zero-day attack traffic shows a different and more severe imbalance. Mirai accounts for 92.9% of the attack samples, with BASHLITE representing only the remaining 7.1%. This asymmetry is directly relevant to the per-family zero-day results presented later in this section, because the overall zero-day catch rate is strongly dominated by the Mirai detection performance, even when BASHLITE behaves very differently. To quantify this directly, we computed the AUROC separately for each family against benign traffic rather than only the pooled value. Mirai alone achieved an AUROC of 0.7295, while BASHLITE alone achieved only 0.5275, close to the 0.5 expected from random guessing. This confirms that the pooled AUROC of 0.6592 is weighted heavily toward Mirai’s larger sample count and that the BASHLITE detection performance is close to statistically negligible on its own.

### 4.1. Known-Class Classifier Comparison

**Figure 5:**
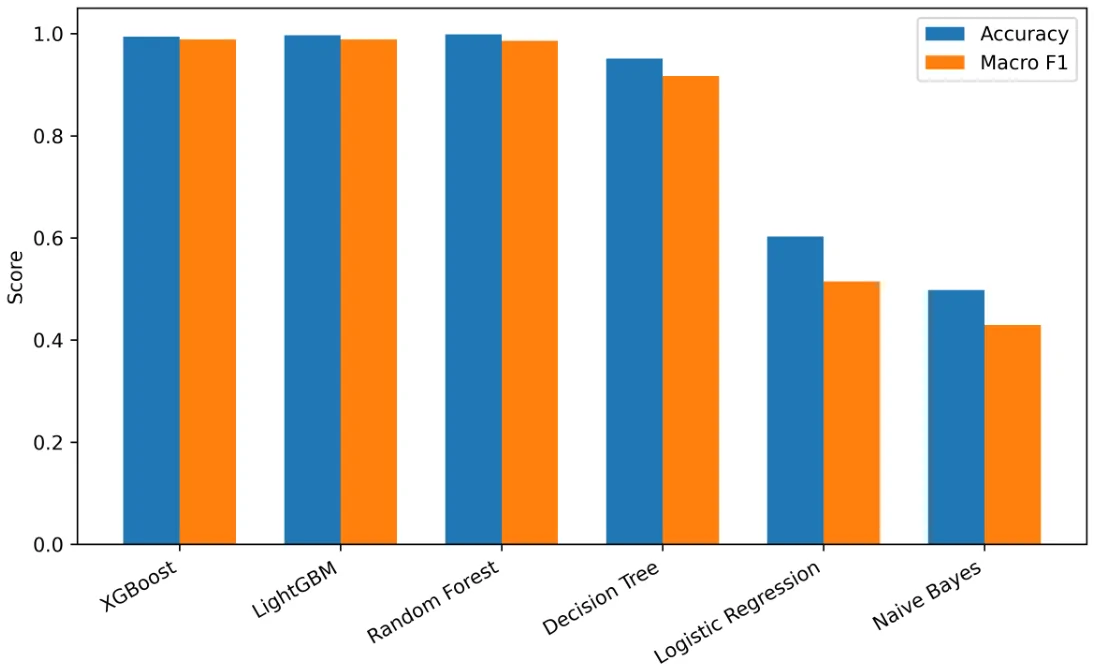
Known-Class Classifier Comparison.

To justify the selection of XGBoost as the Tier 1 classifier, we compared six different classifiers, a single decision tree, and two classical linear and probabilistic models, trained and evaluated under the same conditions on the deduplicated Bot-IoT training and test sets. This ensures that the choice of classifier is justified by direct comparison rather than convention. The comparative results are summarized in Table 3 and Figure 5.

The results show a clear difference between the ensemble tree models and the other classifiers. XGBoost, LightGBM, and random forest all achieved macro-F1 scores above 0.985, and their performance was almost identical, with less than a 0.003 difference between the best and the worst model. In contrast, the single decision tree performed noticeably worse, with a macro-F1 of 0.917. Because it used the same features and the same class weighting, the lower performance suggests that the improvement arises mainly from ensemble learning, not from the feature representation. Logistic regression and Naive Bayes also both performed poorly, with macro-F1 scores below 0.52. This indicates that the relationship between the nine canonical features and the known attack categories is highly non-linear. The traffic patterns appear to involve interactions among multiple features, which tree-based ensemble models can capture more effectively than linear or probabilistic models.

XGBoost was selected as the Tier 1 classifier on this basis. It achieved the best overall macro-F1 score and also offered faster training and inference than random forest. This is relevant for the embedded deployment experiments discussed in Section 4.6.

**Figure 6:**
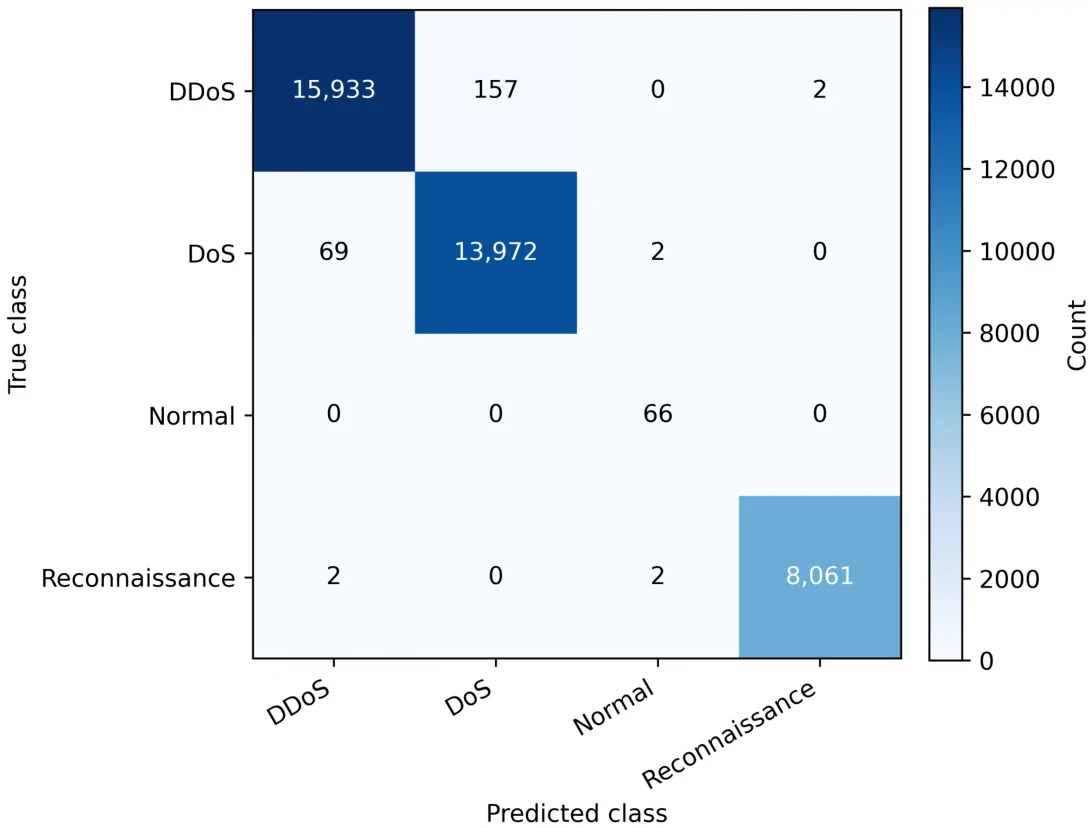
XGBoost confusion matrix on the Bot-IoT test set.

Figure 6 presents the confusion matrix for XGBoost on the held-out Bot-IoT test set. Most of the misclassification occurred between the DDoS and DoS categories. This is expected because both represent high-volume flooding attacks that produce identical traffic statistics in the nine-feature canonical space, particularly in the traffic rate and source packet count. By contrast, the reconnaissance and normal traffic classes are separated from the flooding categories and from each other, with very few errors.

### 4.2. Zero-Day Detection Performance

Table 4 presents the primary zero-day detection results for the MC dropout gate, evaluated against the full zero-day test set described in Table 2. Using the threshold calibrated to a 5% target false positive rate on known traffic, the gate achieved an overall zero-day catch rate of 13.01%. The observed false positive rate of 5.69% on held-out benign traffic was closely aligned with the calibration target. The gate achieved an AUROC of 0.6592 and an AUPRC of 0.9225 for the binary attack-or-benign label.

**Figure 7:**
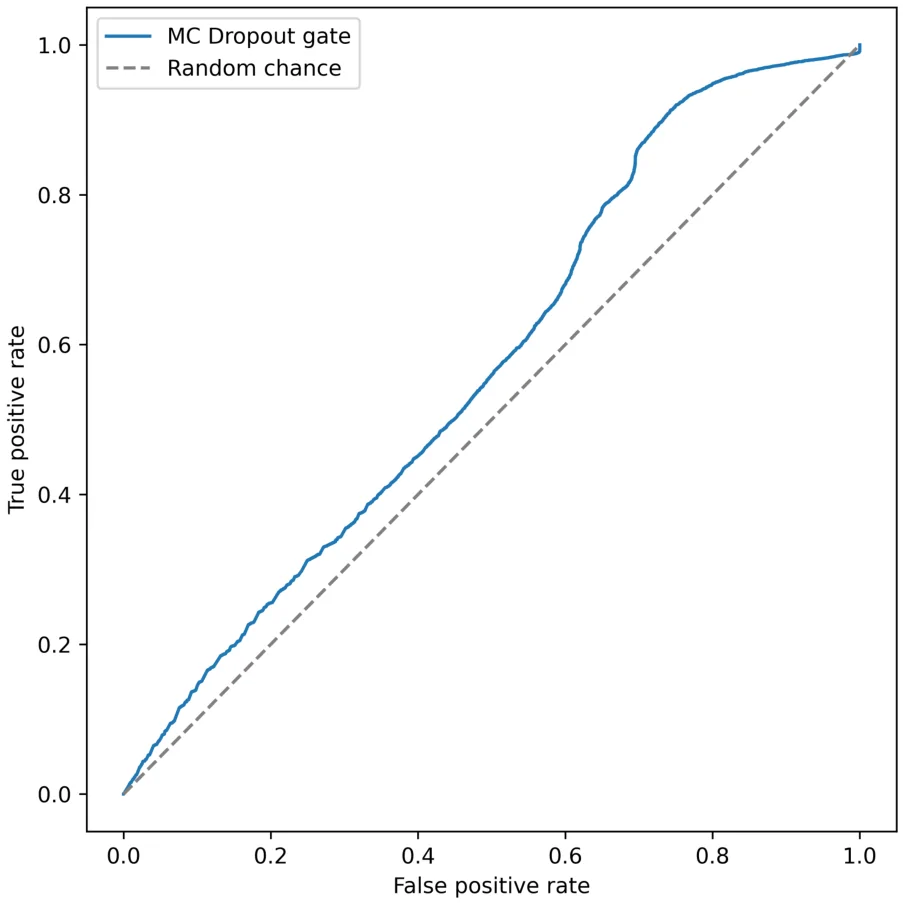
ROC curve for zero-day detection.

Figure 7 shows the ROC curve computed using held-out benign traffic from N-BaIoT and zero-day attack traffic combined. The curve sits only slightly above the diagonal reference line. This is consistent with the AUROC of 0.66 and indicates that the model provides moderate, rather than strong, separation between benign and zero-day traffic.

**Figure 8:**
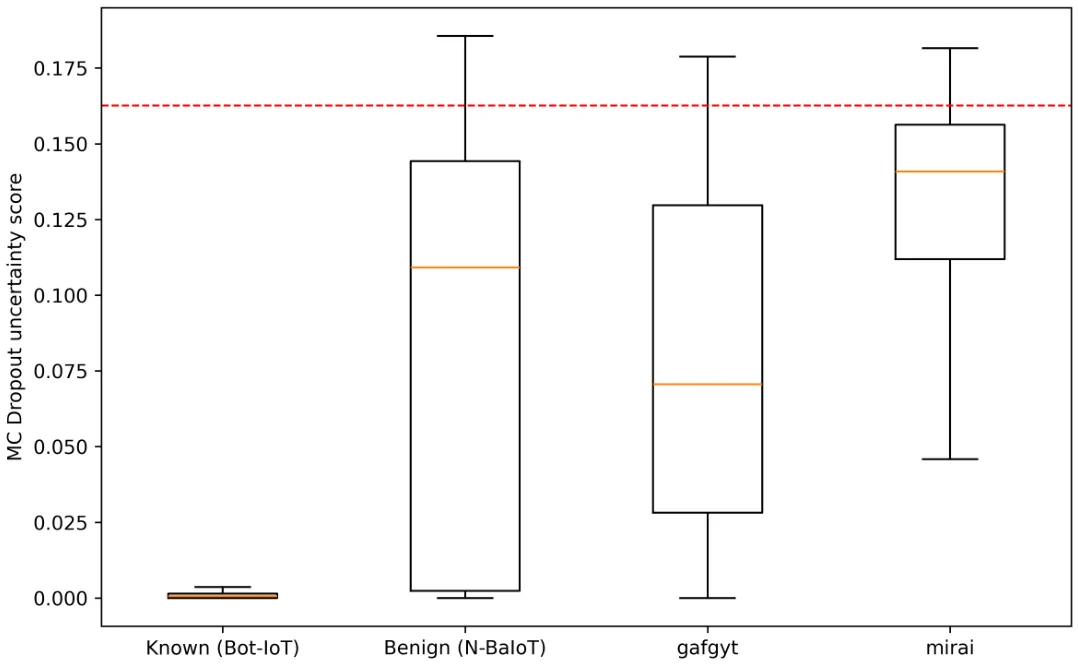
MC dropout uncertainty score distributions.

**Figure 9:**
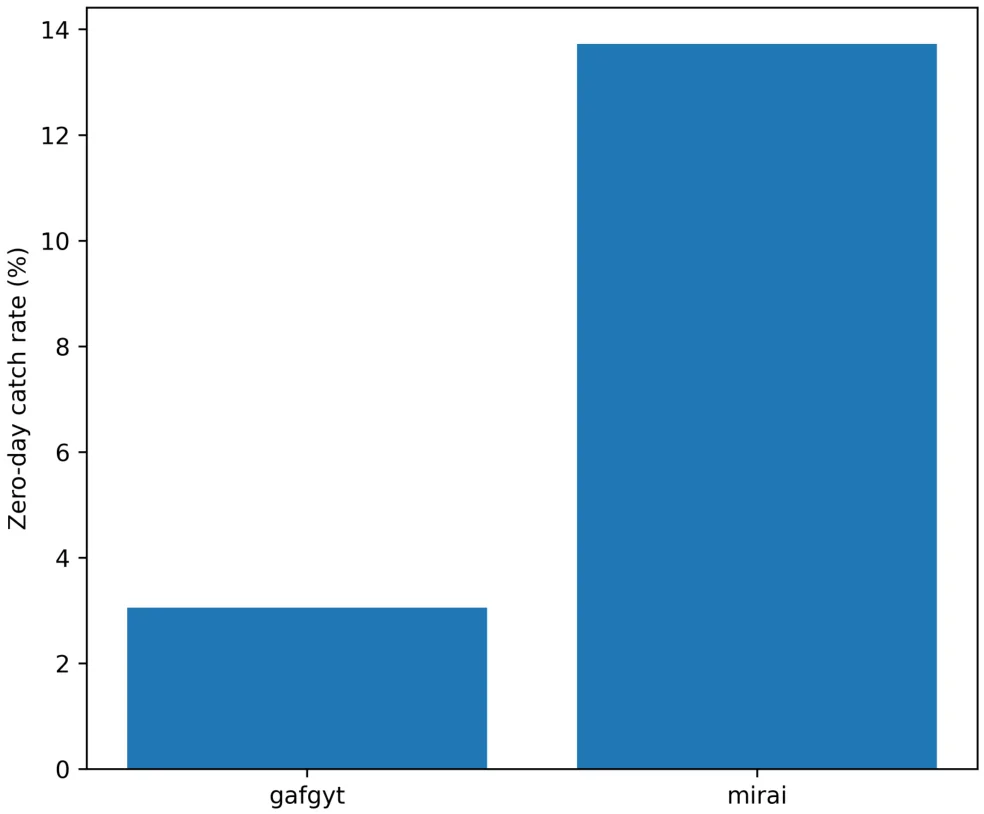
Zero-day catch rate by attack family.

The catch rate differs sharply between the two attack families in the zero-day test set. Mirai traffic was flagged at 13.75%, while BASHLITE traffic was flagged at only 3.24%. Both malware families were entirely unseen during training and calibration and were evaluated under the same calibrated threshold. Figure 8 shows the distribution of the MC dropout uncertainty scores for known Bot-IoT traffic, held-out N-BaIoT benign traffic, and each zero-day attack family. The Mirai uncertainty scores are generally higher than the calibrated threshold for a substantial fraction of the samples. On the other hand, BASHLITE’s uncertainty distribution overlaps heavily with N-BaIoT’s own benign traffic, particularly within the interquartile range. This overlap provides a direct visual explanation for the lower BASHLITE catch rate, as many BASHLITE samples are statistically similar to benign traffic in the current feature representation. Figure 9 summarizes the resulting per-family catch rates.

### 4.3. Comparison with Alternative Methods

**Figure 10:**
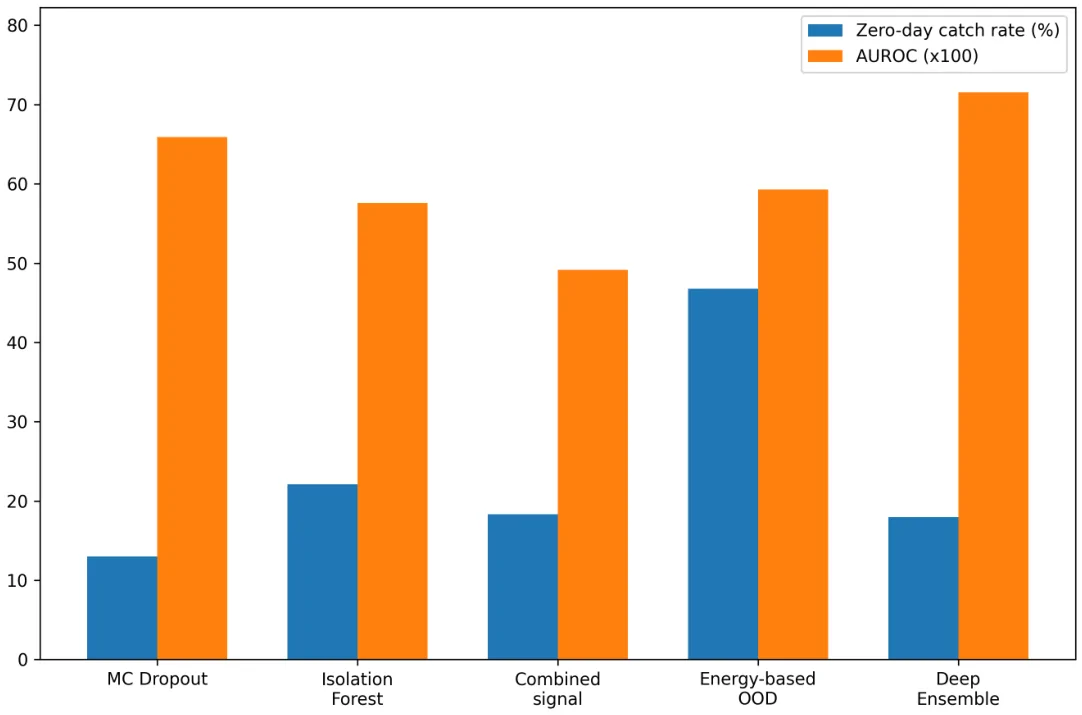
Comparison with alternative methods.

To determine whether the performance of the MC dropout uncertainty gate was mainly due to the uncertainty estimation method itself or was limited by the nine-feature canonical representation, we evaluated the four alternative detection approaches described in Section 3. Both methods were evaluated under the same calibration protocol. This allows a fair comparison of their zero-day detection performance. The results are summarized in Table 5 and Figure 10.

The Isolation Forest baseline achieved a higher zero-day catch rate than the MC dropout gate. However, it produced a lower AUROC. This means that its higher catch rate at the specific calibrated operating point does not indicate stronger detection across all possible thresholds. A particularly important result is that the Isolation Forest catch rate for Mirai was 23.80%, but it was only 0.03% for BASHLITE, which is effectively zero. This pattern can be seen in the MC dropout gate’s own family-level asymmetry as well. The fact that these two different methods show a similar imbalance suggests that the BASHLITE traffic is genuinely difficult to separate from benign traffic in this feature space. Therefore, it is not a weakness specific to MC dropout.

Two further baselines were added to more comprehensively assess the proposed uncertainty gate against the broader OOD detection literature. An energy-based score, computed directly from XGBoost’s own output logits, achieved the highest raw catch rate of any method (46.76%) but a relatively modest AUROC (0.5930), indicating that this high catch rate is concentrated on the easier Mirai family rather than reflecting strong overall ranking quality. A deep ensemble of five independently trained MLP models, using disagreement across models rather than dropout as the uncertainty signal, achieved the highest AUROC of any method tested (0.7152) and was the only method to detect BASHLITE more reliably than Mirai. This result is promising, but it comes at roughly five times the training and inference cost of the single-model MC dropout gate, since it requires maintaining and running five separate networks rather than one. Given this study’s focus on resource-constrained embedded deployment, we retain the single-model MC dropout gate as our primary approach and report the ensemble result as evidence that further stability gains are achievable at an additional computational cost, rather than as a replacement for the proposed method. We did not implement an OpenMax baseline in this study; the energy-based and ensemble baselines mentioned above already span two additional established families of OOD detection methods (confidence/logit-based and ensemble-based), and we consider a distance-based comparison with a method such as OpenMax a direction for future work.

The combined-signal variant, which combined XGBoost prediction confidence with the MC dropout uncertainty score, performed worse than the MC dropout gate alone on every metric. Its AUROC dropped to 0.4916, which is close to random performance. To understand this result directly, we separately evaluated XGBoost’s confidence score on the same zero-day test set, where it achieved an AUROC of only 0.3206. This is below the 0.5 expected of a random classifier. This indicates that XGBoost’s own softmax confidence is not helpful for zero-day status and is actively anti-correlated with true zero-day status. This means that XGBoost tends to be more confident rather than less confident on attack traffic that is found to be a genuinely unseen family. Combining this misleading signal with the MC dropout uncertainty score therefore reduces the quality of the final score instead of improving it. This finding is discussed further in Section 5.

### 4.4. Sensitivity Analysis

To justify the calibrated operating point and the specific hyperparameters used for the MC dropout gate, we examined how the detection performance changed under alternative threshold, dropout rate, and pass count choices.

Table 6 reports the zero-day catch rate at several target false positive rates rather than only the single 5% operating point reported above. The 13.01% catch rate reported in Table 4 corresponds to a conservative operating point; a deployer willing to accept a higher false positive rate can recover substantially more detections, illustrating that the 13.01% figure reflects one specific trade-off choice rather than a fixed ceiling on the method’s capabilities.

We also examined sensitivity to the MC dropout rate, retraining the gate at 0.1, 0.3, and 0.5, in addition to the default 0.2. The AUROC ranged from 0.3672 at dropout 0.1 to 0.6788 at dropout 0.3, with the default value of 0.2 (AUROC 0.6340 on a repeated training run) falling within this range rather than representing an outlier best case. Finally, we examined the number of MC dropout passes used at inference, since this directly affects deployment latency: reducing it from 30 to 15 passes recovers most of the achievable AUROC (0.7325 versus 0.7459), with only a small further loss at 10 passes (0.7212), suggesting that the pass count could likely be reduced for faster inference with a limited cost in terms of detection quality.

### 4.5. Feature Attribution Results

**Figure 11:**
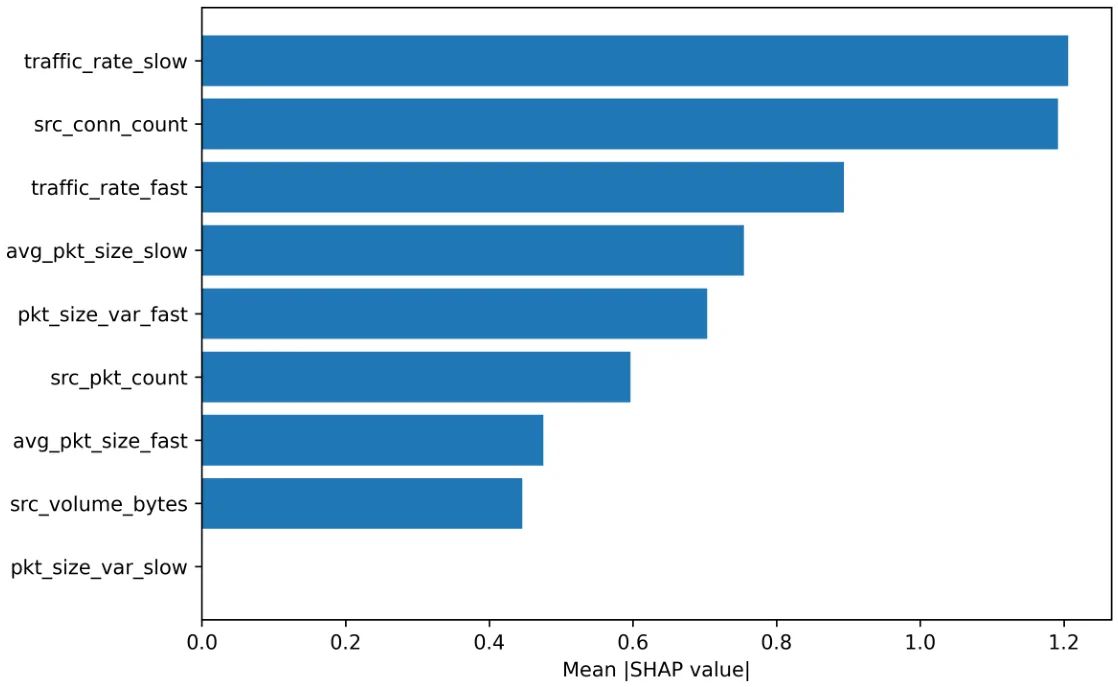
SHAP feature importance for known-class classification.

Figure 11 shows the SHAP feature importance for the XGBoost classifier on the known-class test set. The feature pkt_size_var_slow, which was zero-filled during feature alignment, correctly received zero importance. This indicates that XGBoost learned to ignore this uninformative feature and did not extract any misleading signal from it. Among the nine canonical features, traffic_rate_slow and src_conn_count are the two most influential features for known-class classification as they have the largest SHAP importance values.

**Figure 12:**
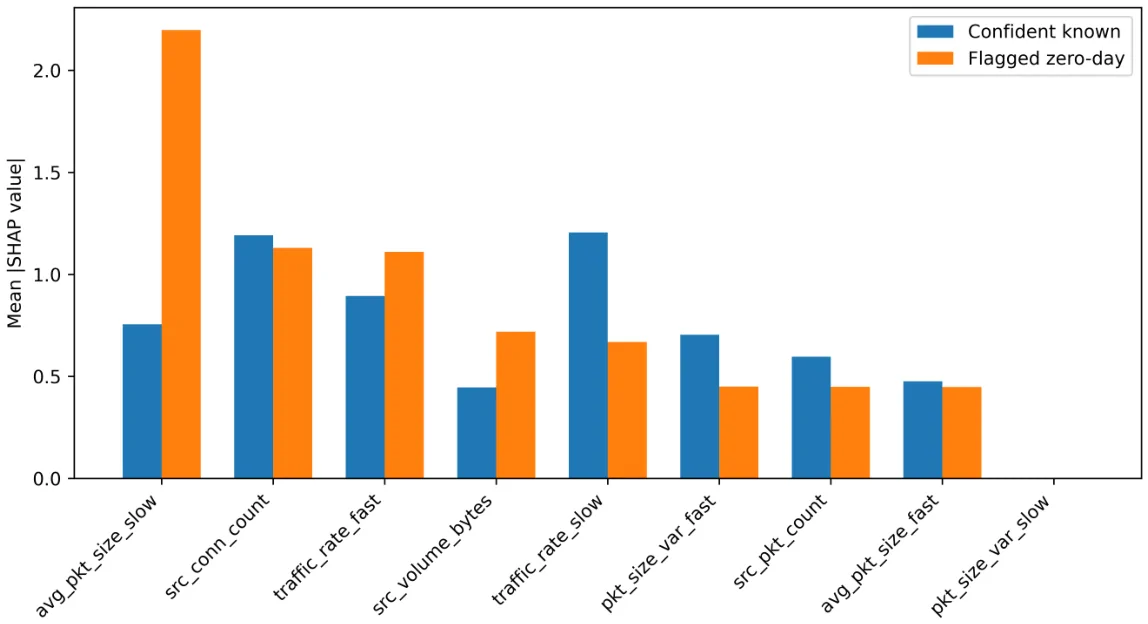
SHAP attribution comparison: confident known versus flagged zero-day traffic.

Figure 12 compares the mean absolute SHAP values for confidently classified known traffic and correctly flagged zero-day traffic. The feature avg_pkt_size_slow shows the largest change among all features. Its mean SHAP value rises from 0.75 for confident known traffic to 2.18 for flagged zero-day traffic, with an increase of 1.42. In contrast, traffic_rate_slow, which is the most influential feature for known-class classification, falls from 1.21 to 0.66 on flagged zero-day traffic. This result suggests that the feature that supports confident known-class decisions is not the same feature driving the model’s uncertainty on unseen traffic. When the gate correctly identifies zero-day traffic, it responds mainly to changes in the longer-window average packet size rather than to the traffic rate signal, which is usually important for ordinary classification. This behavior is consistent with a real difference in packet size behavior between known and unseen traffic, rather than the model relying on artificial or superficial patterns in the data.

The SHAP analysis above explains the known-class classification decisions of the Tier 1 XGBoost classifier specifically and should not be interpreted as explaining the internal uncertainty computation of the Tier 2 MC dropout gate, since the two models are trained independently and perform different tasks. To examine which features drove the uncertainty gate’s own decisions directly, we additionally computed the permutation feature importance on the MLP’s uncertainty score, shuffling each feature’s values in turn and measuring the resulting change in mean uncertainty. This identified pkt_size_var_fast and pkt_size_var_slow as by far the two most influential features for the uncertainty gate, differing from the features identified by the XGBoost-based SHAP analysis above. This confirms that the feature importance for the classification task and that for the uncertainty estimation task are genuinely distinct and that an explanation of one model’s behavior should not be assumed to transfer to that of the other.

### 4.6. Edge Deployment

**Figure 13:**
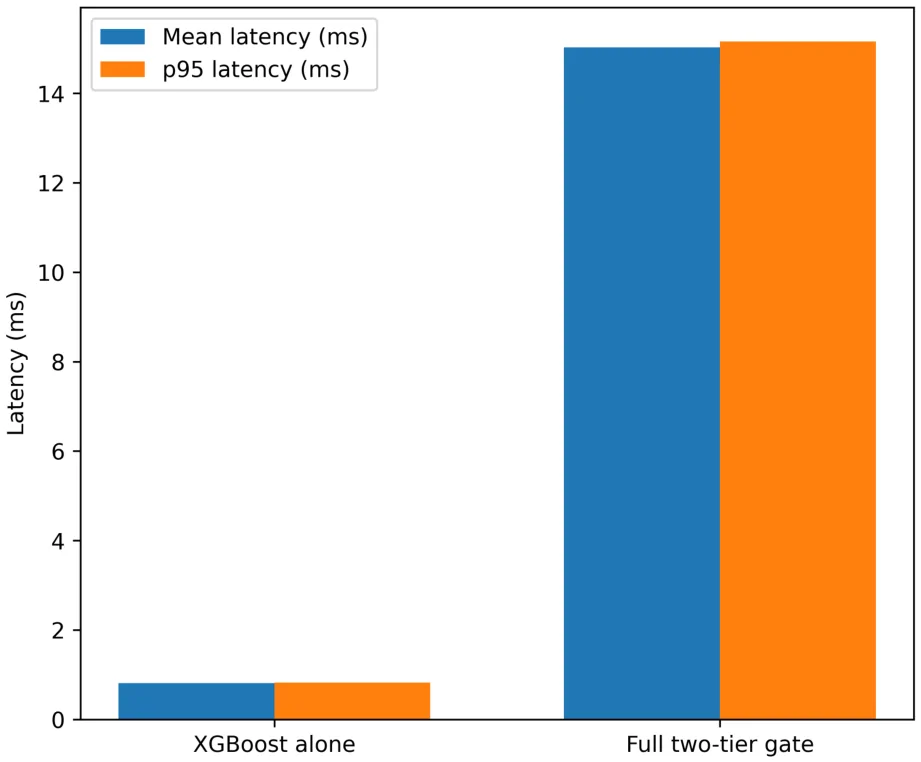
CM5 inference latency comparison.

Table 7 presents the measured inference latency and memory usage for the complete pipeline running natively on the Raspberry Pi CM5. The measurements were obtained using the 2000-sample benchmark set described in Section 3. When XGBoost alone completed inference in a mean of 0.813 ms per sample, the full two-tier pipeline, including all 30 MC dropout stochastic passes, required a mean of 15.031 ms per sample. This is approximately 18 times slower than Tier 1 alone, reflecting the additional computation needed for uncertainty estimation. Figure 13 provides a direct comparison of the two conditions.

Based on the measured mean latency, the full pipeline can process 66.5 samples per second, with a peak memory footprint of 373.5 MB. The higher latency of MC dropout inference was expected as, unlike standard inference, which performs one deterministic forward pass, MC dropout performs 30 sequential stochastic forward passes for each input sample. This additional computational cost is unavoidable, and its impact on the practical deployment of the framework on resource-constrained IoT gateway hardware is discussed further in Section 5.

## 5. Discussion

### 5.1. Discussion

The discussion below focuses on how well the calibrated combination of these methods performs under genuinely cross-dataset conditions. An AUROC of 0.66 for zero-day detection may seem moderate when viewed by itself. However, it should be interpreted against the difficulty of cross-dataset generalization, not against closed-set benchmarks. The heterogeneous benchmark discussed in Section 2 reported cross-dataset F1 scores as low as 0.40–0.56 [4], while many closed-set studies report accuracies above 99%, including the ensemble framework discussed in Section 1 [2]. These results are not directly comparable, since they are obtained only on attack families already present during training. In this study, the model was never exposed to Mirai or BASHLITE traffic during either training or evaluation. The achieved AUROC of 0.66, meaningfully above the 0.5 expected from random guessing, therefore indicates that the model has learned a real zero-day discriminative signal rather than a negative result.

A more important finding is that XGBoost’s own prediction confidence scored an AUROC of only 0.3206 on zero-day traffic, below random guessing, meaning that the signal is not merely unhelpful but points in the wrong direction. This matches well-established findings that modern classifiers, including tree ensembles and neural networks, tend to be poorly calibrated and overconfident on inputs that differ from the training data [16], and it supports the open-set recognition argument that closed-set classifiers cannot reliably distinguish a confident correct prediction from a confident incorrect one [17]. The results in Section 4.2 confirm this directly: XGBoost’s confidence tends to be higher, not lower, on attack families that it has never seen. This is exactly why we treat uncertainty estimation as a separate task and why the combined-signal ablation in Section 4 degraded detection rather than improving it.

The BASHLITE traffic was more difficult to detect than the Mirai traffic across every method evaluated in this study. The MC dropout gate detected only about 3% of BASHLITE, while the Isolation Forest baseline detected almost none of it; in comparison, Mirai achieved catch rates of 13.75% with MC dropout and 23.80% with Isolation Forest. This pattern was consistent across three structurally different detection approaches, a supervised uncertainty gate, an unsupervised anomaly detector, and a combined signal, which suggests that the poor BASHLITE performance is not specific to any one method. The feature attribution results in Section 4 point to a more specific explanation: the uncertainty gate mainly relies on changes in longer-window average packet size to identify zero-day traffic, and Mirai’s traffic pattern produces this shift clearly while BASHLITE’s does not, at least within the nine-feature canonical representation used here. This points to a limitation of the feature space rather than an error in the detection methodology; detecting BASHLITE-style traffic may require additional feature dimensions, such as inter-packet timing jitter, that were not part of the canonical vocabulary constructed in Section 3.

The complete two-tier pipeline achieved mean latency of 15.031 ms on the CM5, about 18 times slower than XGBoost alone, which is directly attributable to the 30 sequential MC dropout forward passes required to estimate uncertainty. This cost is real and should be stated clearly. However, it remains well within the bounds required for practical flow-level network traffic classification, since individual flows are typically aggregated over periods of seconds rather than milliseconds in IoT monitoring systems; a throughput of 66.5 samples per second is unlikely to become a bottleneck for typical IoT edge gateways. Unlike the heterogeneous benchmark discussed in Section 2, which reports inference performance on a server-class GPU [4], the latency and memory figures reported here were obtained on the same type of resource-constrained embedded device that the framework is intended to run on. We therefore believe that these measurements offer a more informative basis for assessing real-world deployability in IoT edge environments than a theoretical feasibility claim. We additionally tested how many stochastic forward passes were actually needed: reducing the number from 30 to 15 passes recovers most of the AUROC benefit (0.7325 versus 0.7459) with an almost identical catch rate, suggesting that the inference latency could be roughly halved with a minimal cost regarding the detection quality on hardware where this trade-off matters.

### 5.2. Limitations and Future Work

This study has several limitations that should be acknowledged. First, the nine-feature canonical vocabulary, while genuinely shared between Bot-IoT and N-BaIoT, is smaller than the native feature space of either dataset; the BASHLITE detection gap discussed above suggests that this constrained representation may be missing features important to some attack families. Second, the Bot-IoT normal class remained very small even after adding benign N-BaIoT traffic to the calibration set, and a larger, more diverse source of known benign traffic could yield a more statistically robust false positive rate estimate. Third, this study evaluated deployment on a single embedded platform; latency and memory characteristics may differ on other ARM-based or resource-constrained architectures that are common in IoT edge gateways. Fourth, several canonical features differ substantially in raw scale between Bot-IoT and N-BaIoT due to their different extraction pipelines; because the detection threshold is calibrated specifically against N-BaIoT’s own benign traffic, the reported catch rates still reflect a genuine within-dataset comparison, although this scale difference remains worthy of further investigation. Two of the nine canonical features also required approximation for Bot-IoT, and post hoc sensitivity testing suggests that one of these may disproportionately affect zero-day detection stability across training runs; refining this feature’s derivation is a direction for future work. Future work could also extend the canonical feature vocabulary using additional timing-based features, evaluate the framework against a third IoT botnet dataset to further test cross-dataset generalization, and explore quantization or model compression strategies that are specifically compatible with MC dropout’s requirement for stochastic inference, since standard post-training quantization pipelines are not designed with this requirement in mind.

## 6. Conclusions

The individual components used in this study, i.e., gradient-boosted trees and MC dropout, are established techniques rather than new algorithms. The contribution of this work lies instead in combining them into a calibrated detection pipeline and testing this pipeline under conditions closer to real deployment than in most prior studies: a genuinely cross-dataset zero-day evaluation, a previously undetected data-leakage problem that we identify and correct, and measurement on physical embedded hardware rather than simulated or server-class estimates. This study examined whether a standard closed-set classifier could serve as a zero-day IoT botnet detector, and each of the following results directly supports the conclusion that it cannot. First, an XGBoost classifier trained on Bot-IoT achieved 99.4% known-class accuracy, yet its own confidence was anti-correlated with genuinely unseen Mirai and BASHLITE traffic from N-BaIoT, producing an AUROC of only 0.32, demonstrating that high closed-set accuracy does not imply reliable zero-day behavior. We therefore introduced an independently trained MC dropout uncertainty gate, which improved zero-day discrimination to an AUROC of 0.66 while maintaining a controlled false positive rate on known traffic. This gap between excellent known-class accuracy and much weaker zero-day discrimination is the central finding of this work, and it indicates that classifier confidence alone should not be trusted as a signal of novelty in IoT intrusion detection, even when it appears accurate on known attack families. The study also identified and corrected a severe train-and-test duplication problem in the original dataset split—a correction that is likely relevant to other studies using similarly structured IoT flow data. The zero-day detection performance was not uniform across attack families: Mirai traffic was significantly easier to detect than BASHLITE traffic for every method evaluated, pointing to a genuine limitation in the shared nine-feature representation rather than a weakness specific to any one detection method. Finally, deployment on a Raspberry Pi Compute Module 5 confirmed that the complete two-tier pipeline operates within practical bounds for real-time IoT network traffic classification. Future work should extend the shared feature vocabulary with additional timing-based features to address the BASHLITE detection gap and evaluate the framework against further IoT botnet datasets to continue testing its cross-dataset generalization beyond the two used here.

## Figures and Tables

**Table 1:** Canonical feature vocabulary and coverage.

Canonical Feature	Bot-IoT Source	N-BaIoT Source	Status
traffic_rate_fast	rate (direct)	MI_dir_L5_weight	Populated
traffic_rate_slow	AR_P_Proto_P_SrcIP	MI_dir_L1_weight	Populated
avg_pkt_size_fast	bytes/pkts (derived)	MI_dir_L5_mean	Populated
avg_pkt_size_slow	TnBPSrcIP/TnP_PSrcIP (derived)	MI_dir_L1_mean	Populated
pkt_size_var_fast	|sbytes–dbytes|/pkts (approximation)	MI_dir_L5_variance	Populated
pkt_size_var_slow	Not available	MI_dir_L1_variance	Zero-filled (Bot-IoT)
src_volume_bytes	TnBPSrcIP	H_L5_weight × H_L5_mean (derived)	Populated
src_pkt_count	TnP_PSrcIP	H_L5_weight	Populated
src_conn_count	N_IN_Conn_P_SrcIP	HH_L5_weight (proxy)	Populated

**Table 2:** Dataset splits.

Split	Rows
Bot-IoT training (known classes)	178,572
Bot-IoT test (known-class evaluation)	38,266
Calibration validation (Bot-IoT val + N-BaIoT benign half)	287,270
Zero-day test (N-BaIoT attacks + remaining benign)	2,013,698

**Table 3:** Accuracy and macro-F1 comparison of known-class classifiers.

Classifier	Accuracy	Macro-F1
XGBoost	0.9939	0.9887
LightGBM	0.9968	0.9886
Random Forest	0.9988	0.9857
Decision Tree	0.9512	0.9169
Logistic Regression	0.6025	0.5149
Naive Bayes	0.4979	0.4294

**Table 4:** Zero-day detection performance.

Metric	Value
Overall zero-day catch rate	13.01%
False positive rate (held-out benign)	5.69%
AUROC	0.6592
AUPRC	0.9225
Mirai catch rate	13.75%
BASHLITE catch rate	3.24%

**Table 5:** Comparison with alternative zero-day detection methods.

Method	Catch Rate	FPR	AUROC
MC dropout gate	13.01%	5.69%	0.6592
Isolation Forest	22.12%	5.35%	0.5759
Combined signal (XGBoost + MC Dropout)	18.33%	5.83%	0.4916
Energy-based OOD (XGBoost logits)	46.76%	5.72%	0.5930
Deep ensemble (5 independent models)	17.95%	5.73%	0.7152

**Table 6:** Zero-day catch rate at different target false positive rates (MC dropout gate).

Target FPR	Achieved FPR	Catch Rate	Mirai/BASHLITE
1%	1.17%	2.72%	2.88%/0.64%
5%	5.78%	11.83%	12.46%/3.54%
10%	11.48%	21.56%	22.62%/7.56%
20%	23.14%	39.34%	41.14%/15.76%

**Table 7:** CM5 edge deployment performance.

Configuration	Mean Latency	p95 Latency
XGBoost alone	0.813 ms	0.826 ms
Full two-tier gate	15.031 ms	15.161 ms

## Data Availability

The Bot-IoT dataset is publicly available from the Cyber Range Lab at UNSW Canberra Cyber (https://research.unsw.edu.au/projects/bot-iot-dataset (accessed on 14 May 2026)). The N-BaIoT dataset is publicly available from the UCI Machine Learning Repository (https://doi.org/10.24432/C5RC8J (accessed on 14 May 2026)). The code used to align, deduplicate, train, calibrate, and evaluate the framework described in this study is available at https://github.com/chinimini532/iot_botnet (accessed on 14 September 2026).

## Abbreviations

The following abbreviations are used in this manuscript:

AUPRC	Area Under the Precision–Recall Curve
AUROC	Area Under the Receiver Operating Characteristic curve
CM5	Compute Module 5
DDoS	Distributed Denial of Service
DoS	Denial of Service
GPU	Graphics Processing Unit
IDS	Intrusion Detection System
IoT	Internet of Things
MC	Monte Carlo
ML	Machine Learning
MLP	Multilayer Perceptron
SHAP	Shapley Additive Explanations
